# Clinical leishmaniosis in a captive Eurasian otter (*Lutra lutra*) in Spain: a case report

**DOI:** 10.1186/s12917-020-02509-x

**Published:** 2020-08-27

**Authors:** Ana Cantos-Barreda, Ricardo Navarro, Luis Pardo-Marín, Silvia Martínez-Subiela, Elena Ortega, José J. Cerón, Fernando Tecles, Damián Escribano

**Affiliations:** 1 Department of Animal Health, Regional Campus of International Excellence “Campus Mare Nostrum”, University of Murcia, 30100 Espinardo, Murcia, Spain; 2 Interdisciplinary Laboratory of Clinical Analysis, Interlab-UMU, Regional Campus of International Excellence “Campus Mare Nostrum”, University of Murcia, 30100 Espinardo, Murcia, Spain; 3 Parque Zoológico Terra Natura, 30100 Espinardo, Murcia, Spain; 4 Department of Animal Production, Regional Campus of International Excellence “Campus Mare Nostrum”, University of Murcia, 30100 Espinardo, Murcia, Spain

**Keywords:** Eurasian otter, *Leishmania infantum*, Leishmaniosis, *Lutra lutra*, Natural infection

## Abstract

**Background:**

Captive and free-ranging wild mammals have been recognized as potential reservoirs of *Leishmania infantum* infection. The aim of this study was to describe the first clinical case of leishmaniosis in the Eurasian otter.

**Case presentation:**

A case of clinical leishmaniosis is reported in a 4-year-old male Eurasian otter housed at a wildlife park (Murcia, South Eastern Spain). The Eurasian otter showed bilateral epistaxis, anorexia, apathy, and weight loss. A complete blood cell count and biochemical analyses revealed hyperproteinemia, hyperglobulinemia, decreases of paraoxonase-1, increases of haptoglobin and ferritin, and proteinuria. Bilateral nephropathy with hydronephrosis, mesenteric lymphadenomegaly, and ascites were also observed. *L. infantum* infection was confirmed by microscopy (amastigotes were detected in macrophages from spleen aspirate), molecular diagnosis (*L. infantum* DNA was detected by real-time polymerase chain reaction), and serology (anti-*Leishmania* IgG2 antibodies were detected by time-resolved immunofluorometry). The animal was treated with allopurinol for 3 months and gained weight, the epistaxis disappeared, and the ferritin concentration decreased.

**Conclusions:**

This is the first report of clinical leishmaniosis in the Eurasian otter. Our results suggest that Eurasian otters are susceptible to infection with *L. infantum* and can develop clinical leishmaniosis in endemic areas.

## Background

Leishmaniosis is a zoonotic disease caused by the protozoan parasite *Leishmania infantum* in the Mediterranean area, affecting a wide range of mammals, including humans [1]. Domestic dogs act as the main reservoir, and *Phlebotomus perniciosus* is the main vector for *L. infantum* in the South East of Spain, an area in which this parasite is endemic [2]*.* Recently, *L. infantum* infection has been described in mustelids such as the domestic ferret (*Mustela putorius furo*) in Spain [3], and free-ranging badgers (*Meles meles*) in Italy [4]. *L. infantum* infection in the Eurasian otter (*Lutra lutra*) was detected for the first time in wild otters from Asturias (North Western Spain) by Oleaga et al. [5]. However, as far as we are aware, no data exist about clinical cases of leishmaniosis in this mustelid, which is a “near threatened” and fully-protected species. The aim of this report was to describe the first clinical case of leishmaniosis in the Eurasian otter.

## Case presentation

**Fig. 1:**
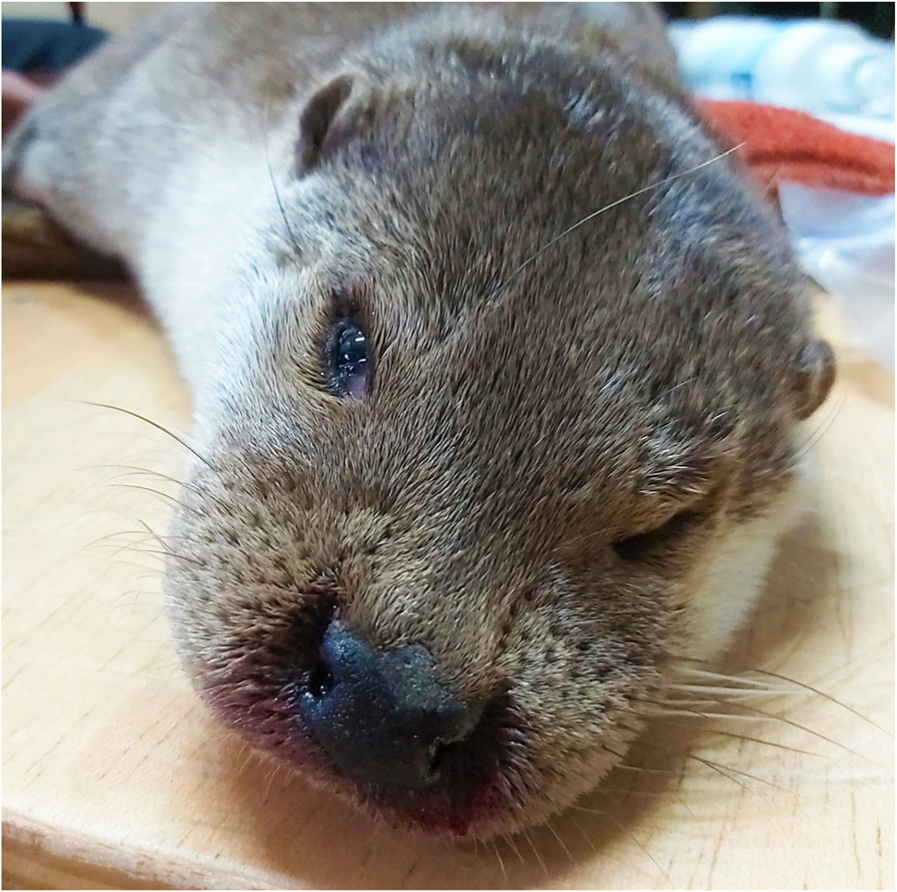
Front view of the Eurasian otter affected by *L*. infantum. Bilateral epistaxis is observed

A 4-year-old male Eurasian otter born in 2015 in the ‘Sendaviva, Natural Park of Navarra’ (42°11′31″N, 1°34′33”O) (North Eastern Spain) was transferred to the ‘Terra Natura’ wildlife park (South Eastern Spain) in 2017. The ‘Terra Natura’ wildlife park is located in the periurban surroundings of Murcia city (38°00′40″N, 1°09′54”O). The otter enclosure consisted of an area with free-access shelters and a pond containing 4 animals of this species (2 male and 2 female). No pour-on anti-sandfly insecticide was applied to the otters. In August 2019 the animal presented bilateral epistaxis, anorexia, apathy, and weight loss (Fig. 1). The animal was sedated prior to manipulation with medetomidine (0.2 mg/kg/i.m.) and ketamine (20 mg/kg/i.m.). Whole blood was taken by venipuncture of the cephalic vein, drained into a 1 ml tube coated with ethylenediaminetetraacetic acid (EDTA) and a 1 ml tube without anticoagulant for a complete blood cell count (CBC) and biochemical analyses, respectively. The CBC was performed in a blood cell counter (ADVIA 120 Hematology System, Siemens, Italy), and the biochemical analyses were performed in an automated biochemistry analyzer (Olympus AU600 Automatic Chemistry Analyzer, Olympus Europe GmbH, Hamburg, Germany). In addition, urine was taken by Ultrasound Guided Cystocentesis for urinalysis. An echography study of the heart and abdominal organs including kidney, spleen, liver, gallbladder, pancreas, and digestive system, was performed. As the biochemical and echographic results were compatible with the clinical leishmaniosis, serological and molecular tests were performed to confirm the presence of the infection.

**Fig. 2:**
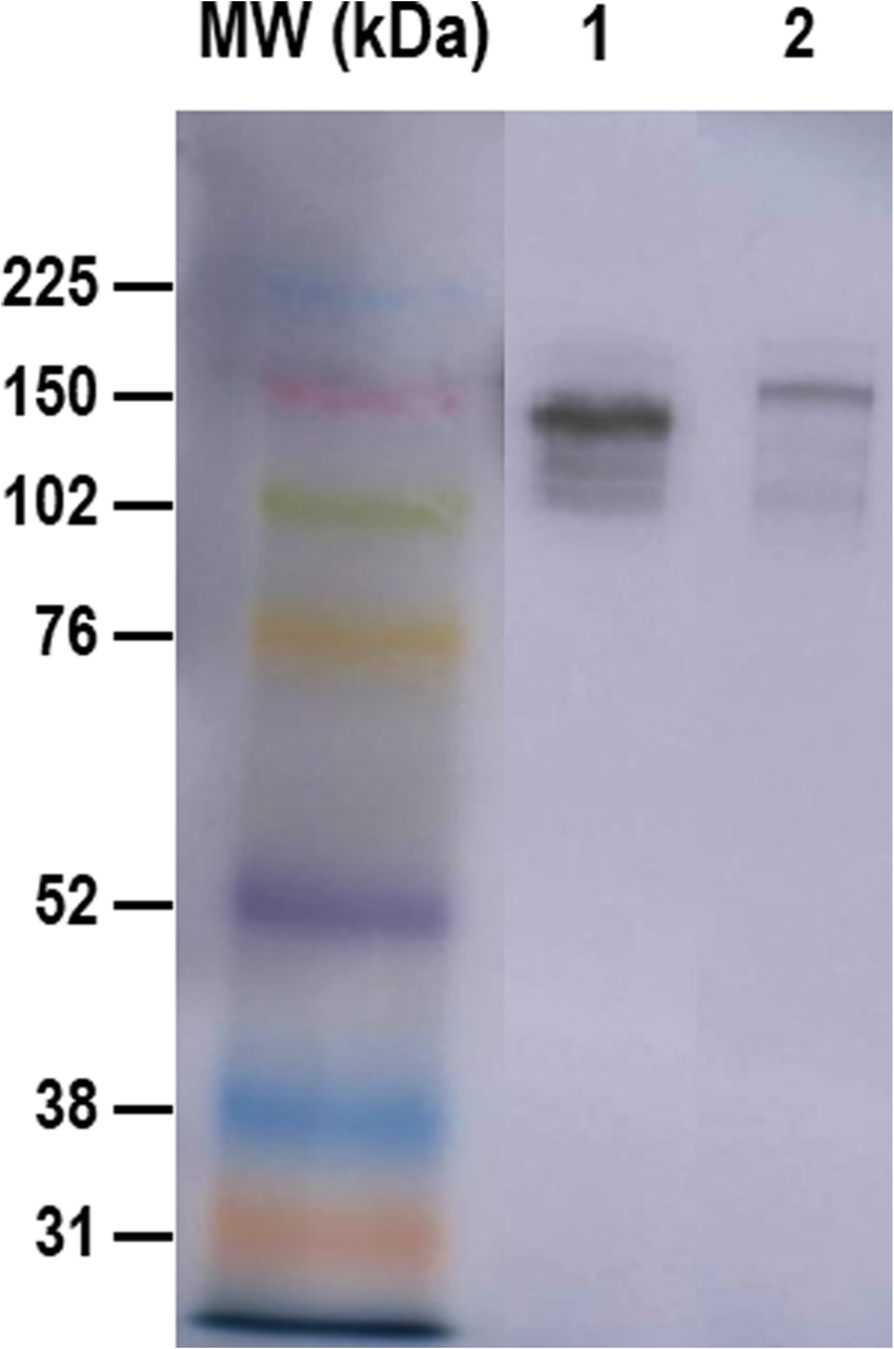
Western blot analysis of IgG2 in the serum of a *Leishmania*-seropositive dog (line 1) and in the serum of the otter affected by *L. infantum* (line 2). A sheep polyclonal antibody anti-dog IgG2 was used. A band of approximately 150 kDa was observed in the dog serum as well as in the otter serum. MW, molecular weight marker (kDa) (Amersham ECL Rainbow Marker, GE Healthcare Bio-Sciences AB, Sweden)

The presence of anti-*Leishmania* IgG2 antibodies was evaluated in the serum of the otter by a time-resolved immunofluorometric assay (TR-IFMA) performed as described by Cantos-Barreda et al. [6]. In order to test that the TR-IFMA assay validated for dog serum can be applied to otter serum, a Western blot analysis was carried out. The Western blot was performed in sera from the otter with leishmaniosis-compatible signs, and from a *Leishmania*-seropositive dog by TR-IFMA. The sera (1:2000 dilution) were separated under reducing conditions on mini polyacrylamide gels (0.1% sodium dodecyl sulfate (SDS), 12% resolving gel, and 4% stacker gel). The resolved proteins were electrophoretically transferred to a nitrocellulose sheet (Bio-Rad, USA) and placed in a ROTI^®^Lumin substrate (Carl Roth, Germany) to block for 2 h at room temperature. Sheep polyclonal antibody anti-dog IgG2 (AHP498; Bio-Rad, USA) at 1:1000 dilution was used as the primary antibody, while rabbit anti-sheep IgG horseradish peroxidase (HRP)-conjugated (SAB3700702; Sigma-Aldrich, USA) at 1:1000 dilution was used as the secondary antibody to detect the bound primary antibody. Signal detection was done using a Pierce ECL2 kit (Pierce, Thermo Fisher Scientific, USA) and was digitalized in the Typhoon 9410 scanner (GE Healthcare, USA). A band of approximately 150 kDa was observed in the otter sample as well as in the canine sample (see Fig. 2 and Additional file 1). These findings corroborated that the TR-IFMA assay using sheep anti-dog IgG2 was able to detect the IgG2 of the otter. The TR-IFMA assay consisted of a non-competitive indirect method based on biotinylated K39 recombinant antigen as a capture reagent, and anti-dog IgG2 polyclonal antibody (Sheep anti-Dog IgG2, Bio-Rad, USA) Eu^3 +^-labelled as a detector. The test included serum from a sick *Leishmania*-seropositive dog as a positive control, and serum from a healthy *Leishmania*-seronegative dog as a negative control. The analysis was performed in a multilabel counter (VICTOR^2^ 1420, PerkinElmer Life and Analytical Sciences, Turku, Finland). Results were expressed as Units of fluorometry for *Leishmania* (UFL). The cut-off was set at 22 UFL. Also, an enzyme-linked immunosorbent assay (ELISA) (Leiscan^®^ Leishmania ELISA Test, Esteve Veterinaria, Laboratorios Dr. Esteve SA, Barcelona, Spain) detecting specific IgG antibodies against *Leishmania* spp. was performed, according to manufacturer’s instructions. Sera samples were diluted 1:20 and the results were expressed as sample-to-positive (S/P) ratio, calculated by optical density (OD) sample/OD low positive control. Values of S/P ratio above 1.1 were considered positives.

The presence of *Leishmania* spp. DNA in whole blood and bone marrow was evaluated by amplification of the kinetoplast DNA sequence of *Leishmania* spp. using an rtPCR with primers and probes previously described [7]. Bone marrow was taken through fine-needle aspiration from the costochondral union, and drained into a 1 ml tube coated with EDTA. DNA was extracted using the High Pure PCR Template Preparation Kit (Roche, Germany), following the manufacturer’s instructions. The rtPCR was performed in the QuantStudio 5 Real-Time PCR System (Applied Biosystems, Foster City, CA, U.S.A.). The internal control TaqMan Exogenous Internal Positive Control Reagents (VIC Probe) (Exo IPC) (Applied Biosystems, CA, U.S.A.) was included. The rtPCR was performed in a final volume of 20 μL, including 1× iTaq Universal Probes Supermix (Bio-Rad, CA, U.S.A), 900 nM of each primer, 200 nM of TaqMan probe, 1× Exo IPC Mix, 1× Exo IPC DNA, and 50 ng of DNA from each sample. The cycling parameters were: 50 °C for 30 s, 95 °C for 10 min, 45 cycles at 94 °C for 30 s, and 55 °C for 1 min. Each amplification run included positive and negative controls, and each measurement was accomplished in triplicate. For the *Leishmania* species identification, the PCR products from the positive samples were purified using the High Pure PCR Product Purification Kit (Roche, Germany) and submitted for sequencing at the Section of Molecular Biology, University of Murcia. The sequences obtained were compared to those available in GenBank.

Additionally, as negative control, the same analyses were performed on a 3-year-old female Eurasian otter from the same wildlife park with no clinical signs compatible with leishmaniosis.

**Fig. 3:**
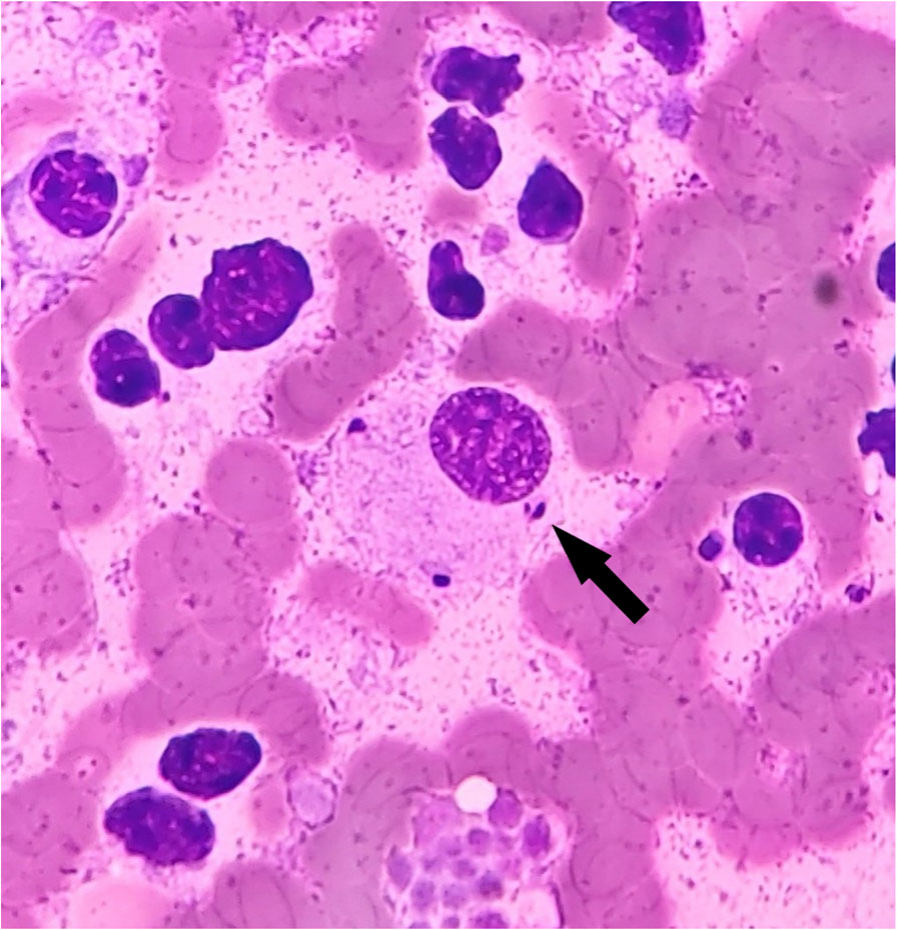
Cytology of spleen of the sick Eurasian otter obtained by fine-needle aspiration. Oval microorganisms with eccentric nucleus compatible with *Leishmania* spp. amastigotes are found into the cytoplasm of macrophages. Note the eccentric nucleus and the kinetoplast of this microorganism (arrow). The slide has been stained with Diff-quick stain and observed at ×100

All the results appear in Table 1. The occurrence of leishmaniosis was confirmed, and specific treatment was administered (allopurinol 15 mg/kg/24 h/p.o.). Treatment monitoring was performed, in November 2019, after 3 months of treatment. Bilateral nephropathy with hydronephrosis, mesenteric lymphadenomegaly, and ascites were observed. The CBC revealed decreases in white blood cells, and decreases in platelets in the *Leishmania*-positive otter. The serum biochemical profile for the *Leishmania*-positive otter showed hyperproteinemia, hyperglobulinemia, decreases of PON-1, and increases of haptoglobin and ferritin. The urinalysis revealed proteinuria and decreases in the osmolarity and specific gravity. The presence of anti-*Leishmania* IgG2 antibodies, and a positive result by rtPCR in terms of whole blood and bone marrow, was observed for the *Leishmania*-positive otter. The sequencing of positive samples confirmed the identification of *L. infantum*. The amplified sequence exhibited 100% similarity with a *L. infantum* reference sequence. In addition, oval microorganisms with an eccentric nucleus compatible with *Leishmania* spp. amastigotes were observed in the cytology of the spleen (Fig. 3).

**Table 1:** Clinical signs, analytical findings, cytology, serology, and rtPCR results recorded in the case of the two Eurasian otters

Animal description	Male, 4 years		Female, 3 years (negative control)
Dates of screening	August 2019 (diagnosis)	November 2019 (treatment)	November 2019
Clinical signs	Bilateral epistaxis, anorexia, apathy, weight loss, bilateral nephropathy with hydronephrosis, mesenteric lymphadenomegaly, ascites	Light hydronephrosis	No signs
CBC (reference values)^a^			
White blood cells (3.1–19.2 × 10^3^/mm^3^)	4.5	**3**	4.6
Platelets (178–777 × 10^3^/mm^3^)	**156**	296	nd
BP (reference values)			
Total proteins (6–7.7 g/dL)^a^	**10.4**	**12.2**	6.7
Albumin (1.25–3.6 g/dL)^a^	2.4	2.1	3.6
Globulin (2.7–4.8 g/dL)^a^	**8**	**10**	3.1
PON-1 (3–4.3 IU/mL)^b^	**2.1**	**2.5**	6.5
Haptoglobin (< 3 g/L)^b^	**3.3**	1.4	0.1
CRP (< 12 μg/mL)^b^	3.7	4	6.7
Ferritin (60–190 μg/L)^b^	**2820**	**266**	111
Blood urea nitrogen (17.3–68.1 mg/dL)^a^	37.1	nd	nd
Serum creatinine (0.7–1 mg/dL)^a^	0.7	nd	nd
Urynalisis (reference values)^b^			
Urine protein/creatinine (UPC) ratio (< 0.2)	**3**	nd	nd
Urine osmolarity (> 500 mOsm/L)	**363**	nd	nd
Urine specific gravity (1.015–1.045)	**1.012**	nd	nd
Serology results by TR-IFMA (< 22 UFL)	**266.6**	**229.4**	4.84
Serology results by ELISA (< 1.1 S/P ratio)	**4.9**	nd	0.3
Spleen cytology (microscopy)	Presence of *Leishmania* spp. amastigotes	Presence of *Leishmania* spp. amastigotes	nd
RtPCR results (parasites/ng DNA × 10^3^)			
Peripheral blood	**13.89**	nd	NAC
Bone marrow	**222.81**	nd	nd

Data of analytes over or under the reference values, and positive tests, are shown in bold. *CBC* Complete blood count; ^a^Hematological and biochemical Eurasian otter reference values [8]; *BP* Biochemical profile; ^b^Interlab-UMU biochemical canine reference values; *PON-1* Paraoxonase-1; *CRP* C-reactive protein; *TR-IFMA* Time-resolved immunofluorometric assay; *UFL* Units of fluorometry for *Leishmania*; *ELISA* Enzyme-linked immunosorbent assay; *S/P ratio* Sample-to-positive ratio; *rtPCR* Real-time polymerase chain reaction; *nd* Not determined; *NAC* No amplification curve

## Discussion and conclusions

To the best of the authors’ knowledge, this is the first report describing a case of clinical leishmaniosis in the Eurasian otter. This species has been reported as being susceptible to *Leishmania* infection, as DNA from this parasite was detected in 7 out of 10 wild healthy Eurasian otters from North Western Spain, a non-endemic area for canine leishmaniosis (CanL) [5]. Santoro et al. [9] confirmed *Ehrlichia canis* and *Rickettsia* spp. infection in the South Italian population of Eurasian otters, and Chadwick et al. [10] indicated that *Toxoplasma gondii* infection is common in Eurasian otters in the United Kingdom. In addition, it was reported that this species may occasionally be infected with *Dirofilaria immitis* [11].

The diagnosis of clinical leishmaniosis described in this report was demonstrated by serological and molecular results, similar to a previous report describing the first case of *L. infantum* infection in a domestic ferret (*Mustela putorius furo*) in Spain [3]. In dogs, the serum total levels of anti-*Leishmania* IgG, IgG1 and IgG2 are elevated during the active phase of *Leishmania* infection, the IgG2 being the predominant subclass [12, 13]. At this moment, elevated levels of anti-*Leishmania* IgG2 in serum were found, as previously reported in dogs with clinical leishmaniosis using TR-IFMA [6]. High levels of anti-*Leishmania* IgG were also detected in the serum from the sick otter by the commercial ELISA. So, both serological results (TR-IFMA and ELISA) were in accordance. The Western blot analysis showed a band of approximately 150 kDa in the otter’s serum as well as in the canine serum, corresponding to the molecular weight of the IgG [14]. These findings support that the TR-IFMA using sheep anti-dog IgG2 is able to detect the IgG2 of otters. These results are in line with those of a previous study in which an indirect enzyme-linked immunoassay using anti-dog IgG detected the IgG of seven marine mammals including sea otters (*Enhydra lutris*) [15].

At the time of diagnosis, the otter presented bilateral epistaxis, anorexia, and apathy, all of which are clinical signs compatible with CanL [1]. The main changes detected in the analytical profile of the *Leishmania*-positive Eurasian otter were also in accordance with those reported in CanL: hyperproteinemia, hyperglobulinemia, decreases of PON-1, increases of haptoglobin and ferritin, and proteinuria [1, 16]. After treatment, the general health condition of the animal improved, as it did not show any epistaxis, and the ferritin concentration decreased, as reported in CanL [16].

Natural infection by *L. infantum* in wildlife species in South-Eastern Spain was described in captive wolves and brown bears, and in free-ranging wildlife species such as foxes, rabbits and rodents [17, 18]. In addition, Muñoz et al. [2] reported sand fly vector abundance in the ‘Terra Natura’ wildlife park. Based on our findings, we could hypothesize that Eurasian otters may act as a secondary reservoir for *L. infantum* infection in the Mediterranean basin. Infection in wildlife carnivores suggests that a sylvatic cycle of this pathogen may occur, being of particular importance to human and pet health [4].

A case of clinical leishmaniosis was, for the first time to the best of our knowledge, detected in a captive Eurasian otter. Therefore, the susceptibility of Eurasian otters to *L. infantum* infection and the development of clinical leishmaniosis in endemic areas should be considered.

## Supplementary information

**Additional file 1. **The original Western blot analysis image for Fig. 2. The target protein analyzed by Western blot was IgG2 in the serum of dogs and otters at dilutions 1:1000 (lines 1–4) and 1:2000 (lines 5–8). Lines 1 and 5: serum from the *Leishmania*-seropositive dog. Lines 2 and 6: serum from the *Leishmania*-seronegative dog. Lines 3 and 7: serum from the *Leishmania*-seropositive otter. Lines 4 and 8: serum from the *Leishmania*-seronegative otter. A sheep polyclonal antibody anti-dog IgG2 was used. A band of approximately 150 kDa was observed in the canine sera as well as in the *Leishmania*-seropositive otter serum. MW, molecular weight marker (kDa) (Amersham ECL Rainbow Marker, GE Healthcare Bio-Sciences AB, Sweden).

## Data Availability

All data generated or analysed during this study are included in this published article.

## Abbreviations

PON-1: Paraoxonase-1
EDTA: Ethylenediaminetetraacetic acid
CBC: Complete blood cell count
UFL: Units of fluorometry for *Leishmania*
ELISA: Enzyme-linked immunosorbent assay
S/P: Sample-to-positive
OD: Optical density
rtPCR: Real-time polymerase chain reaction
BP: Biochemical profile
CRP: C-reactive protein
TR-IFMA: Time-resolved immunofluorometric assay
nd: Not determined
NAC: No amplification curve
