# Supplementary material for: Clinical leishmaniosis in a captive Eurasian otter (Lutra lutra) in Spain: a case report
Source: BMC Vet Res. 2020 Aug 27;16:312. doi: 10.1186/s12917-020-02509-x (PMC7450804; doi:10.1186/s12917-020-02509-x)
Supplement: Supplementary file 1 — Additional file 1. The original Western blot analysis image for Fig. 2. The target protein analyzed by Western blot was IgG2 in the serum of dogs and otters at dilutions 1:1000 (lines 1–4) and 1:2000 (lines 5–8). Lines 1 and 5: serum from the Leishmania-seropositive dog. Lines 2 and 6: serum from the Leishmania-seronegative dog. Lines 3 and 7: serum from the Leishmania-seropositive otter. Lines 4 and 8: serum from the Leishmania-seronegative otter. A sheep polyclonal antibody anti-dog IgG2 was used. A band of approximately 150 kDa was observed in the canine sera as well as in the Leishmania-seropositive otter serum. MW, molecular weight marker (kDa) (Amersham ECL Rainbow Marker, GE Healthcare Bio-Sciences AB, Sweden). [file 12917_2020_2509_MOESM1_ESM.pdf]

Additional file 1

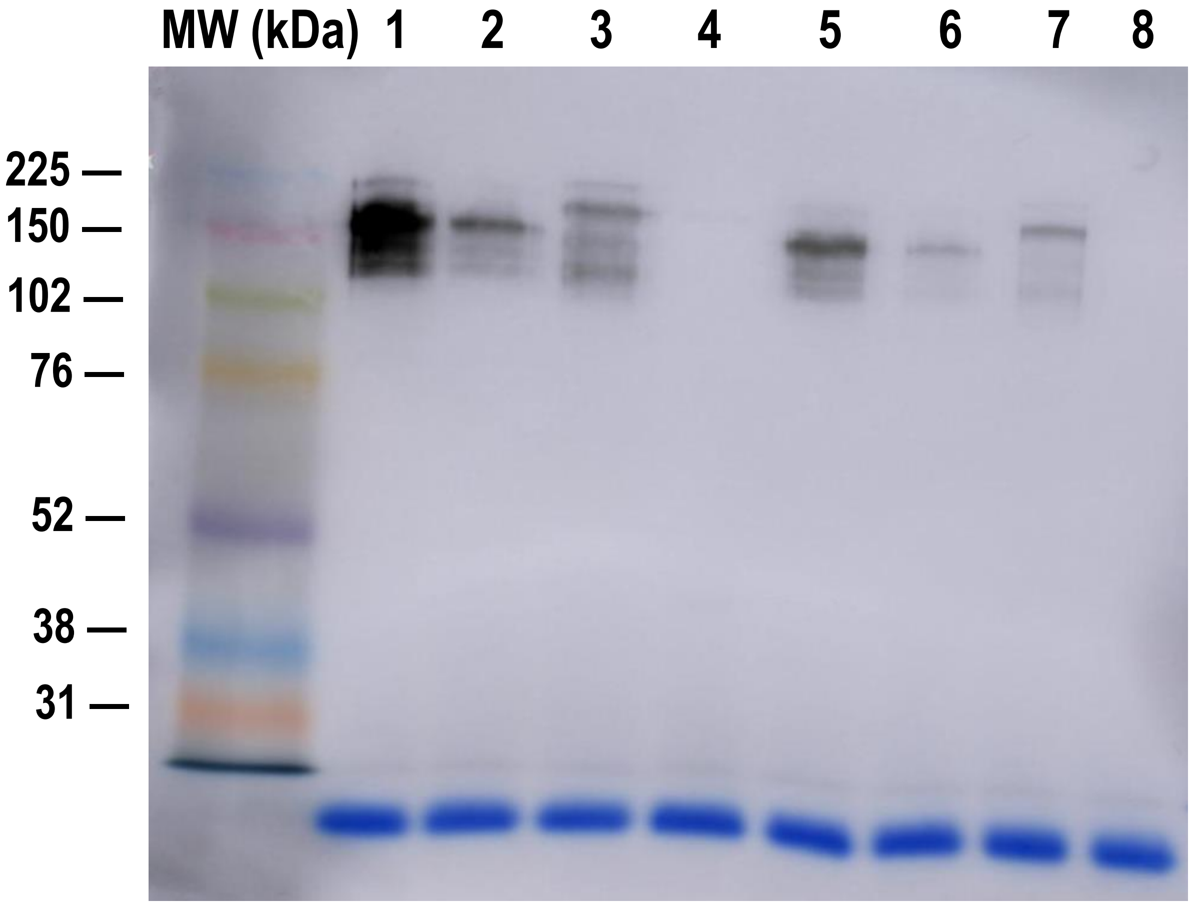

The original Western blot analysis image for Fig. 2. The target protein analyzed by Western blot was IgG2 in the serum of dogs and otters at dilutions 1:1,000 (lines 1-4) and 1:2,000 (lines 5-8). Lines 1 and 5: serum from the *Leishmania*-seropositive dog. Lines 2 and 6: serum from the *Leishmania*-seronegative dog. Lines 3 and 7: serum from the *Leishmania*-seropositive otter. Lines 4 and 8: serum from the *Leishmania*-seronegative otter. A sheep polyclonal antibody anti-dog IgG2 was used. A band of approximately 150 kDa was observed in the canine sera as well as in the *Leishmania*-seropositive otter serum. MW, molecular weight marker (kDa) (Amersham ECL Rainbow Marker, GE Healthcare Bio-Sciences AB, Sweden).
